# Highly sensitive and selective sugar detection by terahertz nano-antennas

**DOI:** 10.1038/srep15459

**Published:** 2015-10-23

**Authors:** Dong-Kyu Lee, Ji-Hun Kang, Jun-Seok Lee, Hyo-Seok Kim, Chulki Kim, Jae Hun Kim, Taikjin Lee, Joo-Hiuk Son, Q-Han Park, Minah Seo

**Affiliations:** 1 Sensor System Research Center, Korea Institute of Science and Technology (KIST), Seoul 136-791, Republic of Korea; 2 Department of Physics, University of Seoul, Republic of Korea; 3 Department of Physics, University of California at Berkeley, Berkeley, California 94720, USA; 4 Molecular Recognition Research Center, Korea Institute of Science and Technology (KIST), Seoul 136-791, Republic of Korea; 5 Department of Electronics and Communications Engineering, Kwang-woon University, Seoul, Republic of Korea; 6 Department of Physics, Korea University, Seoul 136-701, Republic of Korea

## Abstract

Molecular recognition and discrimination of carbohydrates are important because carbohydrates perform essential roles in most living organisms for energy metabolism and cell-to-cell communication. Nevertheless, it is difficult to identify or distinguish various carbohydrate molecules owing to the lack of a significant distinction in the physical or chemical characteristics. Although there has been considerable effort to develop a sensing platform for individual carbohydrates selectively using chemical receptors or an ensemble array, their detection and discrimination limits have been as high in the millimolar concentration range. Here we show a highly sensitive and selective detection method for the discrimination of carbohydrate molecules using nano-slot-antenna array-based sensing chips which operate in the terahertz (THz) frequency range (0.5–2.5 THz). This THz metamaterial sensing tool recognizes various types of carbohydrate molecules over a wide range of molecular concentrations. Strongly localized and enhanced terahertz transmission by nano-antennas can effectively increase the molecular absorption cross sections, thereby enabling the detection of these molecules even at low concentrations. We verified the performance of nano-antenna sensing chip by both THz spectra and images of transmittance. Screening and identification of various carbohydrates can be applied to test even real market beverages with a high sensitivity and selectivity.

There has been growing interest in the label-free detection of molecules and their analysis by optical detection system including plasmonic methodologies^1^, resonant microcavities^2^, optical-fibre sensors^3^, and interferometry-based biosensors^4^. Because an optics-based label-free sensing system can provide quantitative counting and avoid any complexity from fluorescence tagging, terahertz (THz) time-domain spectroscopy (TDS) for molecular detection is also of greatly increasing importance^5^,^6^. The THz TDS is basically a non-contact, non-destructive, and label-free sensing tool suitable for the examination of biological^7^ and chemical substances^8^, especially, for the direct demonstration of intermolecular signatures within the broad THz spectrum^9^. Recently, metamaterial-based THz sensing platforms were introduced in order to increase the detection sensitivity of small molecules or microorganisms^10^. In particular, subwavelength THz metamaterials on the order of λ/10–λ/10,000 can induce a huge field enhancement in transmission^11^. Then, the locally enhanced THz field can be readily used to detect chemical and biological substances with a high sensitivity, because the molecular absorption cross sections are effectively increased by the field enhancement^12^.

Here, we present a nano-slot-antenna array-based THz sensing method supporting the highly accurate measurement of even very small quantities of sugar molecules and the selective identification of different sugar molecules. Our designed nano-antenna allows detecting molecules over a very wide concentration range from hundreds of micromoles to tens of moles. Furthermore, the imaging results for THz transmittance demonstrate the remarkable selectivity working for only the targeted sugar molecule. Finally, we demonstrate the detection of sugar levels for real market beverages including diet sodas known to contain a very small amount of artificial sweeteners, which can lead to a new type of non-contact and non-invasive sensing application including a precise sugar monitoring.

We performed THz TDS of various sugar samples in pellet forms. A commercial THz TDS system (Zomega THz Z-3XL) was used to obtain THz spectra in the frequency range of 0.5–2.5 THz. We measured the THz spectra for several carbohydrate pellets; for D-glucose (a monosaccharide which has the simplest form of a carbohydrate, Fig. 1(a)), fructose (a monosaccharide, Fig. 1(b)), sucrose (a disaccharide which is a combination of the monosaccharides D-glucose and fructose, Fig. 1(c)), and cellulose (a polysaccharide which consists of a linear chain of many linked D-glucose units, Fig. 1(d)). And we plotted the absorption coefficients, 
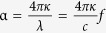
, where *c* is the speed of light, *κ* is the imaginary part of the refractive index and *f* is the *f*requency (Supplementary Information). The hydroxyl functional group has strong absorption peaks, as shown in the figure, e.g. at 1.4 THz, 1.7 THz, and 1.8 THz for D-glucose (Fig. 1(a)), fructose (Fig. 1(b)), and sucrose (Fig. 1(c)), respectively. In stark contrast, the polysaccharide group has no recognizable absorption features in the THz spectrum owing to the complexity of the chains between various structural groups and the lack of coordinated hydrogen-bond vibrations in the crystalline state (Fig. 1(d))^13^.

We suggest a novel type of plot-colour contour mapping of the THz absorption coefficients in terms of the frequency which helps us easily recognize various types of carbohydrates in accordance with their spectral characteristics. A monosaccharide group was mapped using a red tone (mannose, galactose, D-glucose, arabinose, and fructose), a disaccharide group was mapped using a yellow tone (maltose and sucrose), a polysaccharide group was mapped using a green tone (cellulose, glycogen, and amylose), and artificial sweeteners were mapped using a blue tone (aspartame and acesulfame K). The absorption features are well matched with previous study of inter- and intra-molecular vibrational modes of saccharides^14^. We took notice of several pronounced absorption peak positions in the spectra in Fig. 1(e) (white and black dashed circles) and then designed the nano-slot antennas for highly sensitive and selective THz sensing, even after dissolution with a very low concentration of molecules.

A schematic of the nano-antenna experiment is shown in Fig. 2(a). Our designed nano-slot-antenna array can induce strong THz field localization and a very large field enhancement in transmission^11^, thereby effectively increasing the absorption cross section of sugar molecules^12^ (Figure S1 in Supplementary Information). We designed two different nano-slot-antenna arrays for specific sugars: the glucose antenna has a length of *l* = 40 μm (the targeted frequency, *f*_*res*_ = 1.4 THz, is for D-glucose absorption) and the fructose antenna has a length of *l *= 35 μm (the targeted frequency, *f*_res_ = 1.7 THz, is for fructose absorption). In our experiment, nano-antennas are closely placed each other with a number of 1400 in total for glucose antenna (2 mm × 2 mm) and 7830 in total for fructose-antenna (4  mm × 4 mm), which are plenty enough to reduce the possible uncertain errors from a random distribution after sugar solution dropping. Several interesting sugars including D-glucose, sucrose, and cellulose were measured with the glucose antenna at first.

We progressively changed the molecular concentrations of the sugars from 0 to 500 mg/dL (0–27.5 mmol/L), which seems quite reasonable because the concentration of the normal fasting glucose level in blood is 70–100 mg/dL (3.9 to 5.5 mmol/L), whereas that for patients with diabetic symptoms is 100–125 mg/dL (5.6 to 6.9 mmol/L) and above^15^. The transmitted THz spectrum for D-glucose molecules at a concentration of 250 mg/dL on bare Si shows no distinguishable features compared to the spectrum for a bare Si wafer (Fig. 2(b)) because of the extremely small absorption cross section of the molecule at the reliable frequency regime. The nano-antenna with 1.4 THz resonance was applied to detect D-glucose molecules with concentrations varying from 0 to 4168 mg/dL (Fig. 2(c)). A strongly localized and enhanced THz field by nano-antenna resulted in a significant increase in the absorption cross section, making the D-glucose molecules clearly visible. The estimated THz field enhancement here is approximately 50, as found in an earlier work^16^. It is noted that the concentration for this experiment varies from 10 to 4168 mg/dL, which completely covers three orders of magnitude of concentration levels.

We applied the glucose antenna to two other carbohydrates: sucrose and cellulose with same concentration range. The most drastic change in transmittance was observed in the D-glucose measurement among the three samples because the D-glucose molecule has a clear absorption peak at 1.4 THz, whereas the others do not. A small change in the transmittance for sucrose (Fig. 2(d)) is responsible for the small feature at 1.4 THz in the absorption spectrum (Fig. 1(c)), and a rare change in transmittance is observed for the cellulose sample. The maximum values of the normalized transmittance, *T*_*max*_, for D-glucose, sucrose, and cellulose are plotted in terms of the concentration levels with exponential decay fittings (Fig. 2(e)), which have clear sample-dependent decay constants. The decreased transmittances for different samples exactly reflect the absolute values of the absorption coefficients of the molecules in Fig. 1, showing the high performance of our system. The resonance frequency is shifted toward a lower frequency with the value *Δf* as the molecular concentration increases (Fig. 2(f)) with a strong sample dependence as well. The frequency shift can be interpreted as the effect from the surrounding media, and stronger shift with higher concentration is due to the thicker stacking of the molecular cladding.

Further measurements with the nano-antenna array targeted to fructose with the resonance frequency at 1.7 THz (fructose antenna) represent a variety of selections according to specific sugar molecules. A notable drastic suppression in transmittance was observed for the fructose molecule; however, a lower change was measured for the D-glucose molecule (Fig. 3(a)). This verifies that our antenna, specifically designed for a certain sugar molecule, excellently works for only the targeted molecule which has a strong absorption at that frequency, but it is insensitive to other molecules. The performance of the sugar antenna with molecular concentration and sample dependencies is now described with finite-difference time-domain (FDTD) calculations.

Transmittance spectra were calculated for two different samples: each has an absorption peak at 1.7 THz and 1.4 THz, respectively (Figure S2 in Supplementary Information). It is clearly shown that the maximum values in transmittances for the sample possessing an absorption resonance at 1.7 THz is distinctively stronger than that for the other sample possessing an absorption resonance at 1.4 THz (Fig. 3(b)). From the good agreement between FDTD calculations and experimental results, we propose a simple model to explain the molecular-selective detection. The direct transmission of THz light can experience an exponential decay in amplitude while propagating within the lossy cladding medium. Therefore, the change in the peak transmittance can be written as 
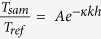
, where *T*_*ref*_ and *T*_*sam*_ are the maximum transmittances through the nano-antenna without and with the cladding; *A* is the transmittance ratio at the air–cladding boundary; *κ* is the imaginary part of the refractive index of the cladding; *k* = 2π/λ is the incidence momentum; and *h* is the cladding thickness, proportional to the molecular concentration. In our simple model, we also assumed that higher concentration means the thicker cladding that yields stronger frequency shift, and we can find that our model provides similar frequency shift behavior as shown in Fig. 3(a,b).

It is discussed that suppression of the peak transmittance strongly occurs when the molecule possesses high absorption at the resonance frequency of the nano-antenna and predicts the suppression behaviour with a thick cladding well (Figure S3 in Supplementary Information). For the frequency shift, when the cladding is sufficiently thin compared to the wavelength of photon, the frequency shift is roughly linearly proportional to the thickness of the cladding, and as the cladding gets thick the shift tend to saturate to the infinitely thick case in previous research^17^.

The high sensitivity and selectivity of our sugar antenna can be also verified by THz far-field imaging in transmittance (Supplementary Information). The fructose antenna was used for the complete discrimination of fructose from D-glucose. The white dashed square lines denote the total nano-antenna area, and the magenta and blue lines represent the dropped stains of fructose (upper-right corner) and D-glucose (lower-left corner) solutions, respectively (Fig. 3(c)). The THz transmittance image at 1.7 THz (Fig. 3(d)) clearly shows different colours between the two sample areas and the slot-antenna-pattern area (middle) owing to different absorptions by the sugar molecules, promising high selectivity for two different sugars. A greater change in colour was observed for the fructose area than the glucose area, as the fructose antenna targeted to.

The fructose antenna was used to detect the sugars contained in various popular sweetened beverages including Coca-Cola Classic, Pepsi-Cola, and Sprite, and in particular, some diet sodas with very low concentrations of sweeteners (Coca-Cola Light and Coca-Cola Zero). The results for the differently decreased transmittances for various beverages showed the clear existence of the sugar content with different concentrations (Fig. 4(a)). We compared the change in the transmittance with the known nutritional values from the manufacturer and a previous report (Fig. 4(b)); for example, Coca-Cola Classic has a sugar content of 10420 mg/dL in total (fructose 6252 mg/dL and 4168 mg/dL glucose)^18^. Because the nano-antenna array is the most sensitive at a concentration level in the range of tens to hundreds of milligrams per decilitre, we specifically focused on the type of diet sodas which have extremely low concentrations of sweeteners such as aspartame and acesulfame K, e. g. tens of mg/dL. The measured large decreases in the transmittances for the two diet sodas are caused by two sweeteners with high absorption features in the range of 1.7–1.79 THz^19^ (Fig. 4(c)). It is notable that our sensing level is valid for monitoring a very small amount of sweetener, even in real market beverages, because these artificial sweeteners have recently received considerable attention owing to their possible addictiveness and toxicity.

In conclusion, nano-antennas operating in the broad THz frequency region can provide highly sensitive and selective detection of carbohydrate molecules, even at concentrations of a few hundred micromoles. Our sugar-antenna-based THz sensing chip exhibits complete selectivity for the targeted sugar molecules, which has the possibility for further applications such as non-invasive blood-sugar monitoring based on blood composition characteristics study. Further, THz imaging using the sugar antenna with two different samples at once strongly demonstrates the high sensitivity and selectivity of the sugar antennas. Finally, the high performance of the sugar antenna was shown with the real market beverages.

## Additional Information

**How to cite this article**: Lee, D.-K. *et al.* Highly sensitive and selective sugar detection by terahertz nano-antennas. *Sci. Rep.* **5**, 15459; doi: 10.1038/srep15459 (2015).

## Supplementary Material

Supplementary Information

## Figures and Tables

**Figure 1:**
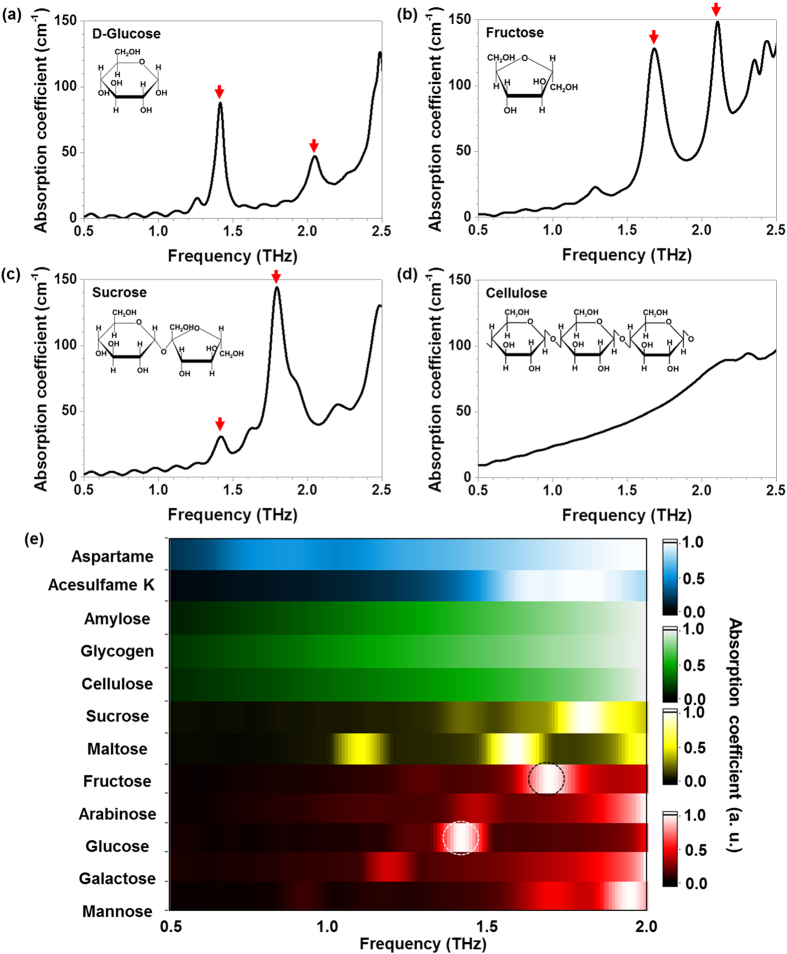
Absorption coefficients for various sugars and sweeteners extracted from THz transmittance measurement. Absorption coefficients at THz frequencies for (**a**) D-glucose (C_6_H_12_O_6_), (**b**) fructose (C_6_H_12_O_6_), (**c**) sucrose (C_12_H_22_O_11_), and (**d**) cellulose ((C_6_H_10_O_5_)_n_) pellets. The insets in (**a**–**d**) show the structural formulas of each saccharide. Distinguishable absorption features appear at 1.4 THz for D-glucose, 1.7 THz and 2.1 THz for fructose, and 1.4 THz and 1.8 THz for sucrose, as marked by red arrows; however, cellulose has no special spectral features. (**e**) Colour contour plots of THz fingerprinting for ten different saccharides and two non-saccharide sweeteners. White and black dashed circles denote the specific frequencies of interest.

**Figure 2:**
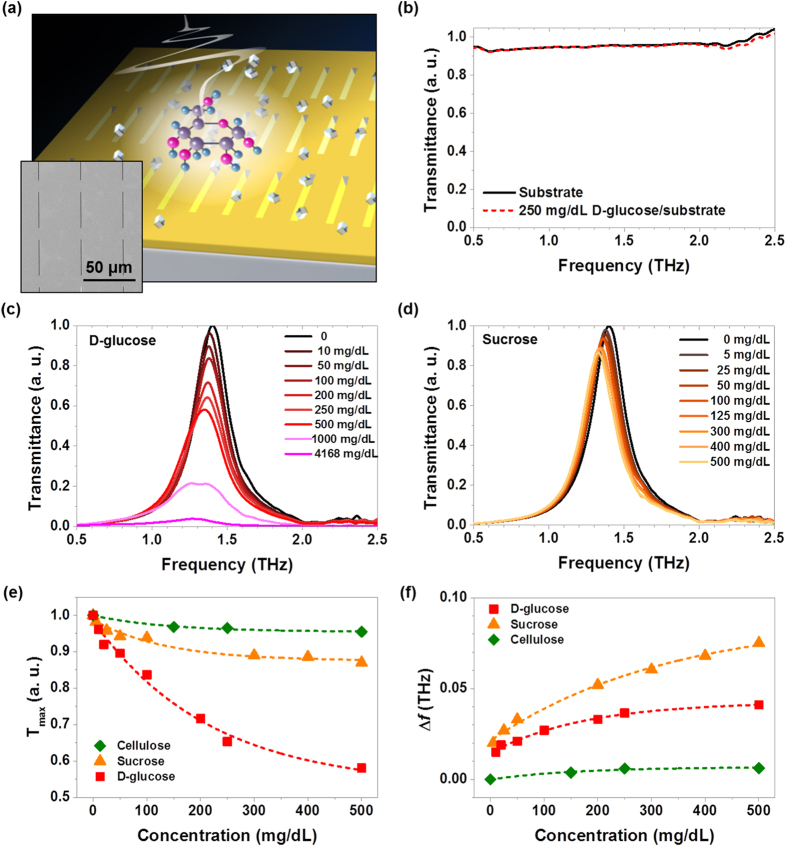
The THz measurements for sugars with and without nano-antennas. (**a**) Schematic of the THz detection of sugar molecules using a nano-antenna array-based sensing chip. The small cubes represent crystallized molecules of sugar and the THz wave is normally incident on the chip. Inset is a microscopic image of the nano-slot-antenna array for fructose. (**b**) Normalized THz spectra measured for a bare Si wafer used as a substrate and 250 mg/dL of glucose on the same Si substrate. (**c**) Normalized THz spectra measured with the glucose antenna for D-glucose and (**d**) sucrose molecules. (**e**) The changes in the maximum values of the normalized transmittances are plotted for D-glucose, sucrose, and cellulose as a function of the molecular concentration level. (**f**) Frequency shifts at the maximum transmittance for three samples are plotted.

**Figure 3:**
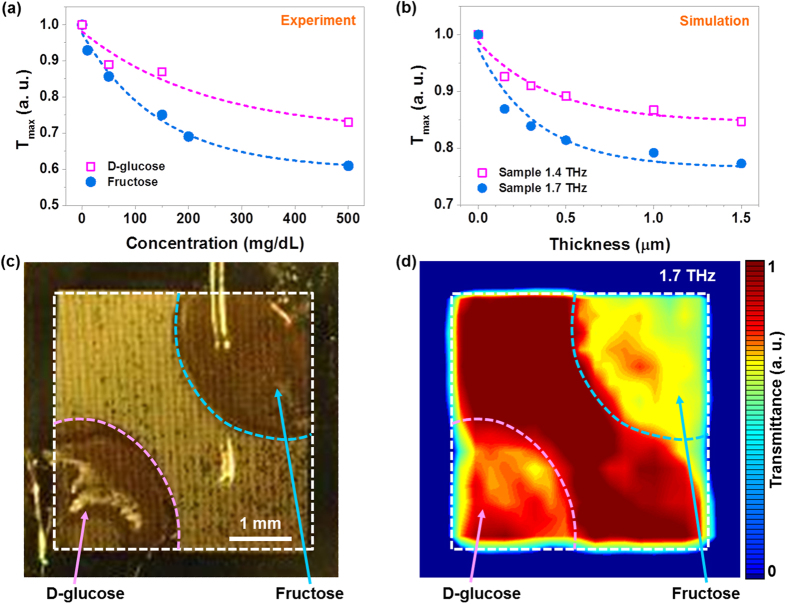
A comparison with THz measurements and FDTD simulations, and THz images obtained with the fructose antenna. (**a**) The changes in the maximum values of the normalized transmittances are plotted for fructose and D-glucose as a function of the molecular concentration level, measured using the fructose antenna. (**b**) Simulation results of cladding thickness-dependent maximum transmittances for two samples having absorption peaks at 1.7 THz and 1.4 THz are shown. The dashed lines are exponential fittings for all cases. (**c**) A photograph of the nano-antenna with 250 mg/dL of dropped fructose (upper-right corner) and D-glucose (lower-left corner) stains. (**d**) A normalized THz transmittance image through the fructose antenna with the two samples.

**Figure 4:**
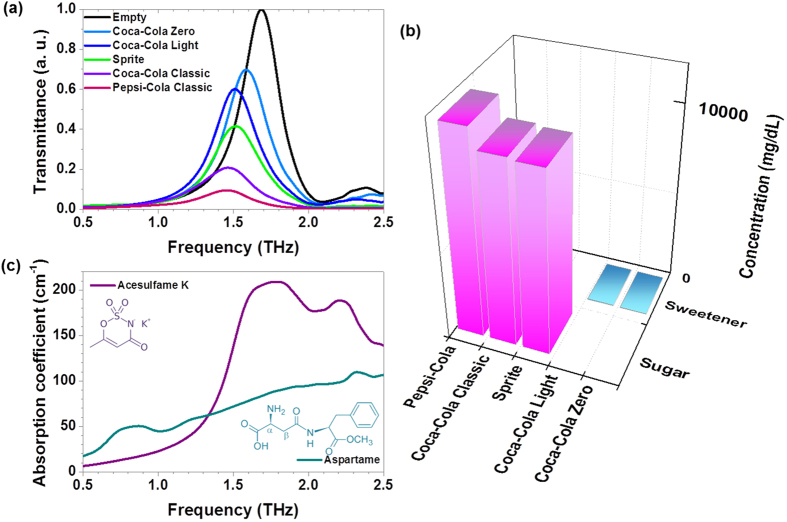
The performance of the THz sugar antenna is verified with real market beverages containing acesulfame K, aspartame, sucrose, fructose, glucose, and so on. (**a**) Normalized THz transmittances using a nano-antenna array with a fundamental resonance at 1.7 THz are shown for Coca-Cola Zero, Coca-Cola Light, Coca-Cola Classic, Pepsi-Cola, and Sprite. (**b**) The total concentrations of sugars and sweeteners in the real market beverages from the manufacturers. (**c**) Absorption coefficients for acesulfame K and aspartame. The insets show the structural formulas.
