# Supplementary material for: Highly sensitive and selective sugar detection by terahertz nano-antennas
Source: Sci Rep. 2015 Oct 23;5:15459. doi: 10.1038/srep15459 (PMC4616020; doi:10.1038/srep15459)
Supplement: Supplementary Information [file srep15459-s1.docx]

Supplementary Information for
Highly sensitive and selective sugar detection by terahertz nano-antennas

Dong-Kyu Lee^1, 2^, Ji-Hun Kang^3^, Jun-Seok Lee^4^, Hyo-Seok Kim^1, 5^_,_ Chulki Kim^1^, Jae Hun Kim^1^, Taikjin Lee^1^, Joo-Hiuk Son^2^, Q-Han Park^6^, and Minah Seo^1†^

^1^Sensor System Research Center, Korea Institute of Science and Technology (KIST), Seoul 136-791, Republic of Korea

^2^Department of Physics, University of Seoul, Republic of Korea

^3^Department of Physics, University of California at Berkeley, Berkeley, California 94720, USA

^4^Molecular Recognition Research Center, Korea Institute of Science and Technology (KIST), Seoul 136-791, Republic of Korea

^5^Department of Electronics and Communications Engineering, Kwang-woon University, Seoul, Republic of Korea

^6^Department of Physics, Korea University, Seoul 136-701, Republic of Korea

^†^ To whom correspondence should be addressed. ^†^mseo@kist.re.kr

1. **THz time-domain spectroscopy and imaging system**

A commercial THz TDS system (Zomega Z-3XL) based on a Ti:sapphire femtosecond laser with a repetition rate of 80 MHz and a wavelength centred at 800 nm is used. The THz-wave source module consisting of a high-voltage-modulated photo-conductive-antenna was used, and an electro-optical-sampling method with a ZnTe nonlinear crystal was used to detect THz waves. THz waves were focused through a TPX (polymethylpentene) THz lens with a size of few millimetres, and samples were mounted at the focal point. Measurements of a sample and reference signal were averaged 10 times, and the entire system was purged with N_2_ gas in a closed box, excluding the Ti:sapphire laser. For the nano-slot-antenna experiments, THz polarization perpendicular to the long axis of the slot was used. The measured transmittance, *T*$=\frac{I_{sam}\left( \omega\right)}{I_{ref}\left( \omega\right)}=\frac{{(E_{sam}\left( \omega\right))}^{2}}{{{(E}_{ref}\left( \omega\right))}^{2}}$ , is obtained from the THz transmittance through a sample attached to an aperture, $I_{sam}\left( \omega\right)={(E_{sam}(\omega))}^{2}$, divided by the THz transmittance through the void aperture, $I_{ref}\left( \omega\right)={(E_{ref}(\omega))}^{2}$. *T* for sugar sample was again normalized to the value through the empty antenna to compare the decreased transmittance ratio.

A far-field THz image in transmission was acquired by moving the sample stage at the THz focal point. The sample was attached to the square aperture having dimensions of 5 mm × 5 mm. A THz transmitted field was obtained for each pixel while the stage was moving with a step size of a 200 µm; thus, the entire image is 25 × 25 pixels. We averaged the THz time-domain waveforms three times and converted them to frequency-domain spectra using a fast Fourier transform (FFT) method, as in typical spectroscopy. Then, a contour colour image of the transmittance in space can be selected at certain frequency. The total data acquisition time was approximately 1.5 h for scanning a 5 mm × 5 mm area, and the entire system was also nitrogen-purged during the measurement.

1. **The THz absorption coefficients and the complex refractive indexes**

The samples consisting of monosaccharides, disaccharides, and polysaccharides were prepared in highly crystalline powder states at first. Then, the samples were thoroughly ground in a mortar and pressed in a pellet die under a pressure of approximately 2000 psi for 5 min. The average thicknesses of the samples are 650 µm on average, and the diameters are 8 mm, whereas the spot size of the THz beam is 2 mm, which is large enough to cover a homogeneous area of the pellets. Such pellet-type samples provide full spectral information including the inter-molecular vibrational modes of the targeted sugar molecules, which allows for full fingerprinting in the THz spectrum (0.5–2.0 THz)^19^.

The refractive indexes for various sugars and sweeteners are extracted from the transmitted THz spectra in the range of 0.5–2.5 THz. The THz time-domain waveforms of a reference and sample were transformed to the frequency-domain using an FFT. The complex optical constants can be calculated with the relationship between the reference and sample waveforms in the frequency domain as follows:

$E_{sam}(\omega)=E_{ref}(\omega)\cdot exp(- \frac{d\cdot\alpha\left( \omega\right)}{2})\cdot exp(i \frac{2\pi}{}n\left( \omega\right)d)$, (1)

where $E_{sam}(\omega)$ is the amplitude of the transmitted signal through the sample, and $E_{ref}(\omega)$ is the amplitude of the input signal through empty space occupied with the sample. $n\left( \omega\right)$ and $\alpha\left( \omega\right)$ are the real parts of the refractive index and absorption coefficient, respectively, and *d* is the thickness of the sample. From the difference between the spectral amplitudes which passed through the sample and reference, the power absorption was extracted, which is related to the imaginary part of the refractive index,$\left( \omega\right)$. $n\left( \omega\right)$ is obtained from the phase difference between the two signals as

$n\left( \omega\right)=1+ \frac{\varphi_{ref}-\varphi_{sam}}{2\pi d}\lambda$, (2)

and the absorption coefficient,

$\alpha\left( \omega\right) =-\frac{2}{d}\ln\left( T \right)= {-\frac{2}{d}ln((\frac{E_{sam}\left( \omega\right)}{E_{ref}\left( \omega\right)})}^{2})= \frac{4\pi k}{\lambda}$, (3)

where $\varphi_{ref}$ is the phase of the reference waveform, $\varphi_{sam}$ is the phase of the signal which passed through the sample, and $\lambda$is the wavelength.

1. **Nano antenna design and sugar molecule dropping**

The nano-antenna arrays consist of two-dimensional void rectangular slots in a 150-nm-thick gold film on a double-side-polished 500-μm-thick silicon wafer fabricated by an e-beam lithography technique. Each rectangular slot antenna has a fundamental resonance frequency, *f_res_*, which has the relation$f_{res}=\frac{c}{\lambda_{res}}=\frac{c}{\sqrt{2({n_{sub}}^{2}+1)} l}$, where *l* is the length of a rectangular slot, and *n_sub_* is the effective refractive index of the substrate^20^. We designed two different nano-slot-antenna arrays for specific sugars: the glucose antenna has a length of *l* = 40 μm (the targeted frequency, *f_res_* = 1.4 THz, is for D-glucose absorption) and the fructose antenna has a length of *l* =35 μm (the targeted frequency, *f*_res_ = 1.7 THz, is for fructose absorption) with the same width *w* = 500 nm for both. Both periods between antennas are 40 μm in the horizontal direction and 50 μm in the vertical direction. Further, these closely placed slot antennas numbering 1400 in total are sufficient to reduce the possible uncertainties from the random distribution of sugar molecules after the sugar solution is dropped.

Aqueous solutions were prepared by dissolving sugar molecules in distilled water at room temperature; then, a 4 µL solution was dropped onto a nano-slot-antenna-array sensing chip by a pipette. We dried the sensing chip at room temperature within a few minutes to remove the broad water absorption at a reliable THz frequency^21^.

1. **Crystallization effect**

Because absorption features appear clearly in crystalline states and ambiguously in amorphous states, the crystallized form is essential for efficient THz sensing. This fact has made it difficult to measure sugar molecules in an aqueous state after dissolution so far. In our experiment, sugar molecules were randomly distributed either on a metal side or at a slot-antenna gap after dropping and the dry process. Scanning electron microscopy (SEM) images, which were taken before (Fig. S1(a)) and after (Fig. S1(b)) THz measurements, show completely different forms of D-glucose molecules. A droplet of the aqueous sugar molecules experienced an amorphizing process, as shown in Fig. S1(a); however, well-crystallized D-glucose molecules inside the nano-slot antenna were formed by a possible THz-irradiation-induced heating effect. The THz-electric-field effect can be also considered to be responsible for the crystallization. It is known that packing of the same molecules with different orientations leads to the formation of a different type of crystal structure during the crystal-growth process^22^. By assisting with the nano-gap structures, the irradiated THz field can act as a monitoring tool for the molecular packing configuration and induce molecular polycrystalline packing at the same time. To the best of our knowledge, this is the first time that the THz-field-induced crystallization process due to the highly aligned electric-field effect has been reported. This crystallized form of sugar molecules inside the nanogap leads to an increased sensing efficiency, even at a very low molecular concentration because the quantitative detection ability indicated in the THz spectra highly relies on the polycrystalline state^23^.

**
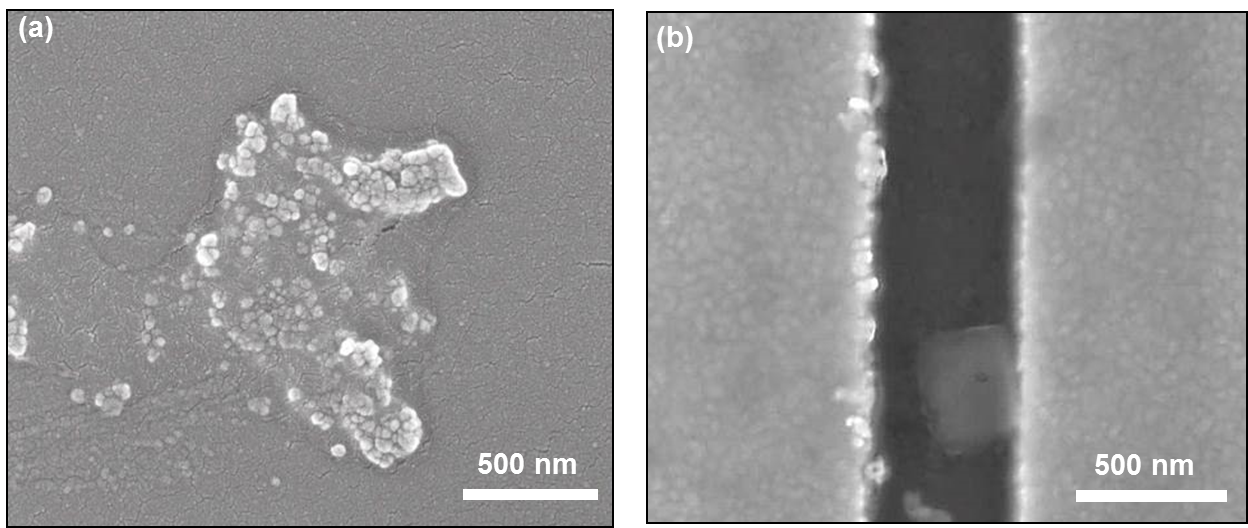
**

**Figure S1.** (a) A scanning electron microscopy (SEM) image of D-glucose molecules on a bare Si surface. (b) An SEM image of crystallized D-glucose molecules inside the nano-slot antennas after THz irradiation.

1. **The complex refractive indexes for sugars and sweeteners**

From the equation (3), the complex refractive indexes for the four sugars and two sweeteners were extracted (Fig. S2).


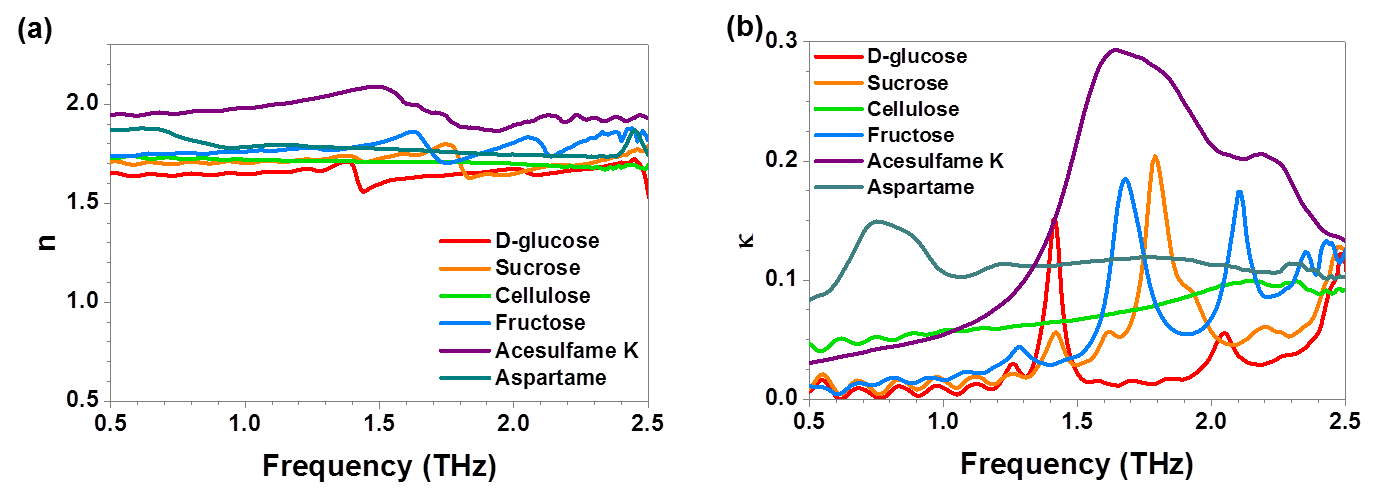


**Figure S2.** Complex refractive indexes for D-glucose, sucrose, cellulose, fructose, acesulfame K, and aspartame are extracted from the THz measurements. The (a) real part, *n*, and (b) imaginary part, κ, of the refractive index are plotted.

For example, the complex refractive indexes for several sugars and sweeteners extracted from the THz transmittance measurements using the specific sugar antenna are summarized in Table S1.

**Table S1. Complex refractive indexes for sugars**

|  | D-glucose  (1.4 THz) | Sucrose  (1.4 THz) | Cellulose  (1.4 THz) | D-glucose  (1.7 THz) | Fructose  (1.7 THz) | Acesulfame K  (1.4 THz) | Aspartame  (1.4 THz) |
| --- | --- | --- | --- | --- | --- | --- | --- |
| *n* | 1.65 | 1.74 | 1.71 | 1.64 | 1.76 | 1.96 | 1.76 |
| *κ* | 0.15 | 0.05 | 0.065 | 0.015 | 0.18 | 0.14 | 0.11 |

*n*: real part of the refractive index

*κ*: imaginary part of the refractive index

1. **FDTD simulations**

The simulations were performed with a nano-antenna which has a fundamental resonance frequency at 1.7 THz, and we assumed that a higher molecular concentration yields thicker cladding deposition around the nano-antenna. In the FDTD calculations, the deposition is simplified as the formation of a uniform cladding on the nano-antenna such that the thickness of the cladding is assumed to be proportional to the molecular concentration which varies in the range of 0–1.5 μm. To mimic molecular-selective detection, we calculated the changes in the THz field transmittances through exemplary nano-antennas supporting the fundamental resonance at 1.7 THz affected by the cladding of two molecular samples possessing different absorption resonances at 1.7 THz and 1.4 THz.

**
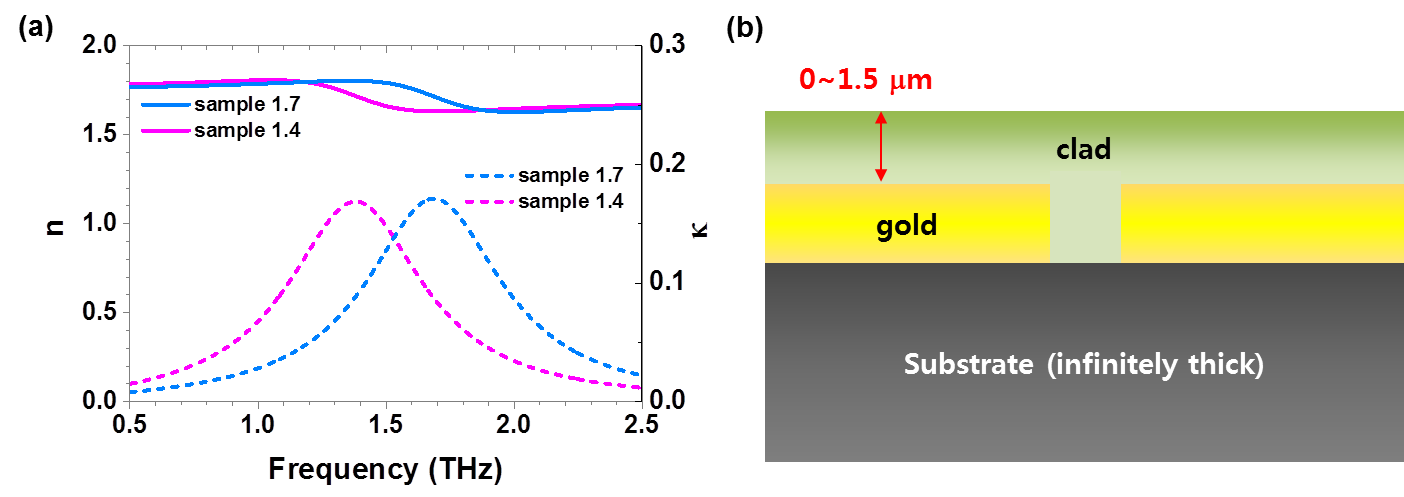
**

**Figure S3.** (a) Complex refractive indexes for two samples used in the FDTD simulations (*n* is plotted on the left axis, and *κ* is plotted on the right axis). (b) Conceptual schematic for the simulation.
